# Susceptibility to a multisensory speech illusion in older persons is driven by perceptual processes

**DOI:** 10.3389/fpsyg.2013.00575

**Published:** 2013-09-03

**Authors:** Annalisa Setti, Kate E. Burke, RoseAnne Kenny, Fiona N. Newell

**Affiliations:** ^1^Institute of Neuroscience, Trinity College DublinDublin, Ireland; ^2^TRIL Centre, Trinity College DublinDublin, Ireland; ^3^Department of Medical Gerontology, Trinity College DublinDublin, Ireland

**Keywords:** McGurk illusion, audio-visual, speech, multisensory perception, semantic, ageing

## Abstract

Recent studies suggest that multisensory integration is enhanced in older adults but it is not known whether this enhancement is solely driven by perceptual processes or affected by cognitive processes. Using the “McGurk illusion,” in Experiment 1 we found that audio-visual integration of incongruent audio-visual words was higher in older adults than in younger adults, although the recognition of either audio- or visual-only presented words was the same across groups. In Experiment 2 we tested recall of sentences within which an incongruent audio-visual speech word was embedded. The overall semantic meaning of the sentence was compatible with either one of the unisensory components of the target word and/or with the illusory percept. Older participants recalled more illusory audio-visual words in sentences than younger adults, however, there was no differential effect of word compatibility on recall for the two groups. Our findings suggest that the relatively high susceptibility to the audio-visual speech illusion in older participants is due more to perceptual than cognitive processing.

## Introduction

Although the human sensory systems are continuously stimulated by multiple sources of information, it is remarkable how the brain efficiently combines the relevant inputs into single objects or events whilst maintaining other sources of information as discrete (see e.g., Calvert et al., 2004). It is known that with ageing, the quality of the sensory inputs diminish due to the degradation of the sensory organs (Fozard and Gordon-Salant, 2001; Gordon-Salant, 2005; Schieber, 2006). Recent research, however, suggests that the ageing brain adapts to these changes to maintain robust perception by relying on the combination of sensory inputs (Laurienti et al., 2006; Peiffer et al., 2007), thus taking advantage of redundancy in cross-sensory information in the environment (Ernst and Bülthoff, 2004). As a consequence, perceptual performance in older persons benefits more from combined inputs than perception in younger adults (Laurienti et al., 2006; Peiffer et al., 2007).

Speech perception is a particularly studied domain in older adults due to its importance for communication and the implications of speech comprehension for social interactions (Pichora-Fuller and Souza, 2003). Since the classic study by Sumby and Pollack (1954) it is well-known that congruent information conveyed across the auditory and visual (i.e., lip-reading) senses facilitates speech perception (Grant and Seitz, 1998; Sommers et al., 2005; Ross et al., 2007; Spehar et al., 2008). In fact visual speech alone can activate the auditory cortex (Calvert et al., 1997). In one recent study comparing younger and older adults, Winneke and Phillips (2011) reported both groups presented reduced P1 and N1 amplitudes in response to audio-visual speech stimuli compared to unisensory stimuli, indicating that fewer resources were necessary to process the audio-visual speech; this effect was more marked in older participants. In addition, both groups showed earlier N1 latency in response to audio-visual stimuli than in unisensory stimuli and the latency shift was related to older adults' hearing thresholds possibly indicating the compensatory function of audio-visual speech to auditory deficits. In fact, older adults, as a consequence of their age-related hearing loss need to rely more on visual speech in order to adequately perceive spoken messages, for example, older adults direct attention toward the speaker's mouth more than younger adults in the attempt to extract sufficient information to support spoken language perception (Thompson and Malloy, 2004) even if lip-reading skills are less efficient in older age (Sommers et al., 2005). While robust evidence shows that older adults benefit more than younger adults of multisensory inputs when speech stimuli are paired with congruent non-speech visual information, e.g., hearing the word “blue” and seeing a blue patch of color (Laurienti et al., 2006), enhanced multisensory integration of audio-visual relative to audio only speech seem not to favor older adults (Sommers et al., 2005; Tye-Murray et al., 2008). This may relate to the quality/integrity of the visual signal as older adults have been shown to have difficulty in processing degraded visual signals (Tye-Murray et al., 2011). For example, Gordon and Allen (2009) showed that older adults benefit from an audio-visual speech input when the level of visual noise is low, however, when the level is high they do not show a benefit possibly because of the difficulty in resolving the visual signal, from the point of view of visual acuity and possibly visual cognitive processing. Nonetheless age-related differences in speech perception appear to be mostly confined to unisensory processing, visual and hearing, while the proportion of benefit obtained in processing auditory speech when a visual signal is added is similar across age groups (e.g., Sommers et al., 2005).

Clearly, both lower level sensory acuity and higher level cognitive processing play a role in older adults audio-visual processing. In fact older adults can capitalize on visual information in speech, when available, but they can also capitalize on the predictability of the semantic content of the message to support comprehension (Pichora-Fuller, 2008; Sheldon et al., 2008). Higher levels of noise can be tolerated when the semantic content of speech is predictable (Pichora-Fuller, 2008). Indeed when older adults cannot rely on the semantic predictability of a sentence, for example, because the sentence does not express a meaningful content, they benefit more from the addition of visual information to auditory speech than younger adults (Maguinness et al., 2011).

The studies mentioned above utilize either auditory only or congruent audio-visual inputs, in the present study we aimed to assess whether audio visual interactions in speech depend on the semantic content of the spoken message utilizing the McGurk effect. McGurk and MacDonald (1976) reported that auditory syllables (e.g., |ba|) when dubbed over an actor visually articulating a different syllable (e.g., “ga”) gave rise to the illusory speech percept of “da.” In the first experiment we utilize the “McGurk illusion” to assess whether older adults show enhanced multisensory integration compared to younger adults, and in the second experiment we manipulate the semantic context preceding the illusory audio-visual combination to assess whether the reliance of older adults on semantic predictability determines the susceptibility to the illusion. We hypothesized that older adults would be more susceptible to the illusion than younger adults due to higher susceptibility to multisensory interactions related to non-pathological unisensory decline (but see Tye-Murray et al., 2010).

Factors that affect susceptibility to this illusion have been widely studied (Campbell, 2008 for a review). For example, susceptibility seems to be independent of facial identity, audio-visual co-location and synchrony (Green et al., 1991). Furthermore, the McGurk illusion has been used as an experimental paradigm to investigate efficient audio-visual integration in different populations (de Gelder et al., 2003; Rouger et al., 2008; Bargary et al., 2009). Cienkowski and Carney (2002) previously used the McGurk illusion to compare audio-visual integration across normally hearing older and younger adults. The younger adults comprised two groups; one group was presented with the same auditory stimuli as the older adults whereas the other group was presented with degraded (i.e., with added noise) auditory stimuli, which rendered their auditory perception “equivalent” to the older group. Although all three groups were susceptible to the “McGurk illusion” there was no overall difference across groups in the frequency of reported illusory percepts. However, the lack of a group difference with the particular stimulus set used (the same two syllables repeatedly across the experiment) does not preclude that older adults may show enhanced integration when words as used, as it is known that differences in complexity across syllables, words and sentences as speech units give rise to different perceptual and cognitive processing as shown by the lack of substantial correlation between performance with these different stimuli (see e.g., Sommers et al., 2005).

In the following experiments we investigated susceptibility to the McGurk illusion as a measure of efficient cross-sensory speech perception in older and younger adults. For the purpose of our experiments we used words (Dekle et al., 1992) rather than syllables as stimuli (Alsius et al., 2005). The use of words represents, in our opinion, a more ecological context to the study of multisensory integration of incongruent speech in older adults. The word stimuli contained the relevant phoneme and viseme combination (e.g., |bale|; [gale]) designed to elicit the illusory speech percept (i.e., “dale”). Our paradigm differed, therefore, from previous studies on speech perception which typically measured the benefit of congruent visual inputs on auditory speech (Grant and Seitz, 1998; Grant et al., 1998; Sommers et al., 2005; Tye-Murray et al., 2008). By using incongruent AV stimuli, we can investigate the extent to which speech perception is robust in older adults in an unreliable speech contexts, in which what is heard and what is seen are incongruent.

Speech comprehension has been shown to be dependent on the efficiency in which auditory (speech) and visual (viseme) speech-related information is integrated by the brain. The “McGurk” illusion has recently been extensively used as a tool to investigate how inefficient audio-visual integration is related to impaired speech perception in both the neurotypical population (Jiang and Bernstein, 2011) and in individuals with neural deficits (Woynaroski et al., 2013). Finally, illusions such as the “McGurk” can reveal wider deficits in information processing beyond speech processing (Woynaroski et al., 2013) and therefore offer a powerful, and engaging, tool for the researcher to investigating the processes more “higher-level” functions.

In sum, we hypothesized that older adults would be more susceptible to the McGurk illusion than younger adults (Experiment 1). We also investigated whether a higher occurrence of McGurk illusions in older adults may depend on higher level processing, such as expectations based on semantic context, or lower level perceptual processing. To that end, in Experiment 2 we manipulated the semantic context of an audio-visual sentence such that sentence meaning was either compatible with the combined illusory percept, either of the unisensory components, or both the fused and unisensory components of a target word embedded in the sentence (Windmann, 2004). This allowed us to assess whether expectations based on the semantic context of the sentence play a role in the number of illusions perceived (Windmann, 2004; Ali, 2007) or if the illusion was perceived in a bottom-up, mandatory way irrespective of semantic context (Sams et al., 1998). We predicted that if the illusion was dependent on higher level cognitive processes such as semantic expectations, as it has been suggested that older persons are particularly dependent on semantic context for speech (Pichora-Fuller, 2008), then the frequency of the illusion should be modulated by the relationship between the semantic content of the sentence and the target word more so in older than in younger participants.

## Experiment 1

### Method

#### Participants

The final sample for this study was constituted by 26 adult volunteers: 13 younger (mean age of 22 years, *SD* = 4) and 13 older (mean age of 65.5 years, *SD* = 4) adults. There were 5 male participants in both the younger and older adult groups. All older adults were living independently in the community and were recruited through the Technology Research for Independent Living (TRIL) project (www.trilcentre.org; see Romero-Ortuno et al., 2010 for a characterization of the TRIL cohort).

A larger group of older participants took part in the study as part of a multisensory perception battery of assessments (*n* = 37). Due to the nature of the study, comparing younger and older participants on processing of speech words and sentences, the need to match younger and older on years of education arose. Education has a pervasive effect on cognitive performance and cognitive decline (Stern, 2009) and therefore on language processing. Among our participants 11 had primary education only; 9 had only 2–3 years of secondary education (inter-certificate or other certificate); 12 had secondary education and 4 had college level education or beyond, for 1 participant the education was unknown. Participants with primary-only and intermediate-secondary level of education were excluded as all the younger sample of age >18 had secondary education. That lead to a sample of 16 participants but an appropriate match to younger participants in regard of sex and education was found for 13 of them.

All older participants retained in the final sample had a Mini Mental State Exam (MMSE; Folstein et al., 1975) score higher than 26 (mean = 29, *SD* = 1) indicating normal cognitive function. Vision was either normal or corrected-to-normal (logMAR test mean = 0.05, *SD* = 0.05). Hearing abilities, as assessed through a Hughson Westlake test with Kamplex BA 25 Screening Audiometer, was normal for their age range. Specifically, participants' mean hearing loss at frequency of 3000–4000 Hz was 16.5 dB (*SD* = 15) in the left ear and 15 dB (*SD* = 14) in the right ear. All younger participants reported normal hearing and either normal or corrected-to-normal vision.

The experiments reported here were approved by the St. James Hospital Ethics Committee and the School of Psychology Research Ethics Committee, Trinity College Dublin and conformed to the Declaration of Helsinki. Accordingly, all participants provided informed, written consent prior to taking part in the study.

#### Stimuli and materials

To create all the stimuli used in the experiment we originally recorded 58 videos [in order to extract 33 visual words (3 repetitions) and 33 auditory words (5 repetitions)] of a female speaker pronouncing a single word. The “McGurk” stimuli were 33 audio-visual incongruent combinations were either taken from a previous stimulus set (Bargary et al., 2009) or were created based on previous literature (e.g., Windmann, 2004) and were known to induce the McGurk illusion (see Table A1). The stimuli were created from digital, audio-visual recordings which were taken using a JVC high band digital video camera in a quiet room with natural light illumination. Each audio-visual stimulus was edited using Adobe Premiere® and had duration of, on average, 1 s. The sound was played at 75 dB.

The audio-visual words articulated by the actor were first separated into the audio and visual components to create speech word stimuli which were either auditory only (A-clear/V-degraded), visual only (V-only), AV-congruent or AV-incongruent words (Bargary et al., 2009). Two additional combinations were created to use as practice. In the A-only condition the words used as auditory stimuli were presented together with a masked (i.e., pixelated) version of the corresponding viseme which effectively blurred the visual information but did not remove it (pixilation: average of 6 pixels in the horizontal axis—from ear to ear—and 12 in the vertical axis—from chin to end of forehead-). In the V-only condition the viseme was presented with the auditory word which was masked using white noise. Therefore, although sound was present, it was not related to the speech signal in any way. For the “McGurk illusion” condition, 33 audio-visual combinations were created by combining an incongruent visual word and auditory word such that the time of the lip movements was manually synchronized with the onset and offset of the auditory word by the use of Adobe Premiere®.

#### Design

The experiment was based on a within-subjects design with the main presentation conditions being either unisensory or multisensory: the two unisensory conditions were A-only and V-degraded and two multisensory conditions were AV-incongruent and AV-congruent. Trials in each condition were presented in separate blocks with four testing blocks in total. Block order was counterbalanced across the entire sample of participants, with the exception of the AV-congruent block which was always presented at the end of the experiment to avoid any effects of congruent word meaning on illusory percepts.

#### Procedure

Participants were seated in front of a desktop computer with their chin comfortably positioned on a chin-rest at 57 cm from the computer screen. They were informed that they would hear and see an actor pronouncing words and that their task was to report the speech word the actor articulated. The reported word responses were directly recorded by the experimenter onto an electronic file.

At the beginning of each trial a fixation cross appeared at the center of the screen for 700 ms followed by the presentation of the speech word stimulus (A, V, or AV—incongruent and congruent conditions). Participants initiated each trial by pressing the spacebar and there was no time limit for responding.

### Results

To assess the task difficulty, we first considered the percentage of trials to which a response was provided by each of the participant groups (i.e., whether correct or incorrect or no response was provided), in each condition (see Table 1). In the Aclear/V-degraded condition, the percentage of trials responded to by the older and younger adult groups was 95.3 and 97%, respectively. The V-only condition was considerably more difficult: older and younger participants responded to only 59 and 72% of the trials, and the mean number of trials to which a response was not provided was 8.8 (*SD* = 9) and 10.8, (*SD* = 11), respectively. This difference reflects the relative difficulty that older people have in lip-reading relative to younger adults. There was considerable variation across participants such that some participants attempted to respond to the majority of trials whereas others responded to none or very few trials. In the AV-incongruent condition (McGurk illusion) the percentage of trials to which a response was provided by the older and younger adults, was 92.5 and 98.6%, respectively. In the congruent AV-condition both older and younger participants responded to all trials. The percentage of words correctly reported was then calculated across participant groups for each condition. The mean percentage of correct responses to the V-only condition was 4.5% (*SD* = 5.4) and 15% (*SD* = 26.3) and to the A-clear/V-degraded condition it was 48% (*SD* = 7%) and 51% (*SD* = 8%) for the young and older adult groups, respectively. It is worth noting that the relatively low number of correct A-only responses may be due to the fact that the visual image is only blurred not absent therefore increasing the probability of multisensory interactions in this condition (MacDonald et al., 2000). There was no difference in accuracy between the two groups on the percentage of words correctly reported in either the V-only [*t*_(1, 24)_ = 1.47, *p* = 0.15] or the A-clear/V-degraded [*t*_(1, 24)_ = 1.12, *p* = 0.27] conditions. In the AV-congruent condition the average percentage of correct responses was 82 and 80% for the older and younger adults, respectively, and there was no difference in performance across the groups [*t*_(1, 24)_ = 0.53, *p* = 0.6].

**Table 1:** **Percentage of responses provided and correct responses**.

	**% responses provided older adults**	**% responses provided younger adults**	**% correct older adults**	**% correct younger adults**
A-clear/V-degraded	95.3	97	15	4.5
V-only	59	72	51	48
AV-congruent	100	100	82	80

The reported speech words to the AV-incongruent condition were classified as either “McGurk-fused,” “McGurk-viseme,” “correct-auditory,” or “other” responses according to the following criteria: responses to the AV-incongruent stimuli were categorized as “McGurk-fused” response when the reported word corresponded to the fused response; “McGurk-viseme” responses occurred were when the participant reported the visual component of the AV word stimulus; “correct auditory” responses occurred when the participant correctly reported the auditory component (i.e., non-illusory percept); and “other” responses occurred when the participant reported a word that did not correspond to any of the other categories. An example of a “McGurk-fused” response is if the auditory word |bale| when paired with the viseme [kale] produces the reported word of “gale.” The “other” category included words which were, for example, similar in phonetics to the auditory word but could not be considered as “McGurk-fused” responses as the place of articulation was the same or similar for the auditory-component of the AV stimulus and the reported word, not intermediate between the place of articulation of the visual and the auditory inputs, as expected in a “fused” response. For example, if the AV combination of |bale| and [kale] gave rise to the unexpected response “bane” this was classified as “other.” This “other” category also included unrelated words (e.g., |pin| – [tin] was reported as “elf”).

Within the AV-incongruent condition, the percentage of reported words categorized under each of these four response types across older and younger participants was: “correct-auditory,” 35 and 37%; McGurk-viseme 7 and 6%; McGurk-fused 37 and 27%; and “other” response was 21 and 29%, respectively.

There were no differences across groups in the “correct-auditory” [*t*_(1, 24)_ = 0.63, *n.s*.] and “McGurk-viseme” [*t*_(1, 24)_ = 0.63, *n.s*.] conditions. In the “McGurk-fused” condition older participants produced significantly more fused responses than younger participants [*t*_(1, 24)_ = 3.04, *p* < 0.01]. The number of reported words which were classified as “other” was significantly higher in younger than in older adults [*t*_(1, 24)_ = 2.8, *p* < 0.01]. Furthermore, the overall number of “other” responses was greater than previously reported. One potential reason for this discrepancy may be that different regional accents or languages may influence the extent to which the McGurk illusion is experienced (see e.g., Sekiyama and Tohkura, 1991; Colin et al., 2005).

In order to test whether the effect of group held across different stimuli, we conducted a by item repeated measures ANOVA with proportion of fused responses per item in each group as within items factor. This factor was significant [*F*_(1, 32)_ = 6.66, *p* < 0.05], with older adults producing on average a higher proportion of fused responses than younger adults.

In order to assess whether younger and older adults might present different patterns in the responses classified as “other,” we conducted further analyses on their types. We found that overall the responses were quite diverse (103 different word types were reported in total across younger and older participants). We classified these responses according to whether they were presented at the same place of articulation as the auditory component of the AV-incongruent stimulus (that is, influenced by the auditory input), as the visual input, or a completely unrelated word. The pattern of response was similar across younger and older participants: we found no difference in the proportion of viseme- (14.75 and 8.22%, respectively) or auditory- (34.4 and 36.9%, respectively) influenced responses across younger and older groups (χ^2^ = 2.09, *p* = 0.14). The majority of words in this category were unrelated to either the auditory or viseme of the AV stimulus (with 50.8 and 54.8% of these words provided by the young and older adults, respectively). This shows that while the regional accent of the speaker might have influenced the responses more in younger than in older, there is no specific difference in the kind of “other” responses provided across age groups, and therefore not reflective of a decision bias across the groups.

### Discussion

These results show that older participants are more susceptible to the McGurk illusion than younger participants with spoken words. In particular, susceptibility to this illusion appeared to stem from multisensory integration rather than a change in unisensory dominance: group differences existed for the McGurk-fused conditions but not for the McGurk-viseme condition. Moreover, we found no difference across groups in their performance to the unisensory (i.e., A-clear/V-degraded and V-only) conditions. This lack of difference could be due to the fact that the older adults in this study are relatively high performing individuals from a convenient sample of generally healthy older volunteers (www.trilcentre.org). Importantly older adults in this sample are highly educated and the level of education is generally associated with hearing (e.g., Agrawal et al., 2008), and cognition (e.g., Stern, 2009).

## Experiment 2

### Introduction

Evidence of an effect of semantic context on the McGurk illusion has previously been provided in two studies on younger adults (Windmann, 2004; Ali, 2007). However, in an earlier study an effect of context was not found (Sams et al., 1998) although methodological differences might be the cause of this discrepancy, for example, Sams et al. (1998) used only one kind of auditory-viseme combination, while others presented more varied stimuli (Windmann, 2004). Nevertheless, both of these studies contained methodological advantages which Ali (2007) subsequently adopted in her study. Specifically, Ali manipulated the compatibility of sentence meaning with either the fused word or the unisensory components and reported fewer illusions if sentence meaning was incompatible with the fused percept. None of the previous studies on the role of sentence meaning on susceptibility to the McGurk illusion, however, compared conditions in which either the fused percept or either of its unisensory components were compatible/incompatible with sentence meaning or both. These additional conditions are required to understand whether participants are simply relying on semantic context, i.e., by the semantic compatibility therefore vastly responding in agreement with the compatible percept, or a more bottom-up fusion between the sensory inputs irrespective of context meaning is maintained.

We expected that if speech perception in older adults is driven more by top-down processing than younger adults then their responses should be more dependent on sentence meaning than those of younger participants. As in Experiment 1, we also expected that older participants would experience more illusions overall than younger adults.

### Method

#### Participants

See Experiment 1.

#### Stimuli and materials

The stimuli were digital audio-visual recordings of a female actor articulating sentences. We followed the same procedure as described in Experiment 1 to make these recordings. For the purpose of this experiment, we created 10 target words by pairing together each of 10 auditory words (e.g., |bait|) with one of 10 visemes (e.g., [gate]) in order to produce incongruent word pairings which were most likely to induce the McGurk illusion (e.g., “date”). We then embedded these target words into sentences. For each AV-incongruent word combination we formulated six sentences, and in each of which we manipulated sentence meaning in the following manner. The meaning of the sentence was compatible with either (1) the illusory “McGurk” (“McGurk-fused”) percept only, (2) the “McGurk-fused” plus A-clear/V-degraded component, (3) the McGurk-fused plus the V-only component. In the remaining three sentence conditions, the meaning was not compatible with the McGurk-fused percept but was also compatible with one of the unisensory components only, i.e., (4) A-clear/V-degraded or (5) V-only. In the final sentence condition (6) meaning was not compatible with any of the components of the word, fused, or unisensory. Table 2 provides an illustration of these six sentence conditions based on the specific example of the auditory word |bait| and viseme [gate] pairing.

**Table 2:** **Example of manipulation of sentence meaning for the audio-visual combination of |bait| and [gate] perceived as** “**date.**”

Target word	Stimuli	Sentence	Compatibility
McGurk-only	Auditory	The teenage boy was looking forward to his bait	No
	Visual	The teenage boy was looking forward to his gate	No
	McGurk fusion	The teenage boy was looking forward to his date	Yes
McGurk and Auditory	Auditory	The couple arranged to meet on a bait	No
	Visual	The couple arranged to meet on a gate	Yes
	McGurk fusion	The couple arranged to meet on a date	Yes
McGurk and Visual	Auditory	The fisherman organized his bait	Yes
	Visual	The fisherman organized his gate	No
	McGurk fusion	The fisherman organized his date	Yes
None	Auditory	My phone number was bait	No
	Visual	My phone number was gate	No
	McGurk fusion	My phone number was date	No
Visual-only	Auditory	To catch a trout I need the bait	Yes
	Visual	To catch a trout I need the gate	No
	McGurk fusion	To catch a trout I need the date	No
Auditory-only	Auditory	The bull was locked behind the bait	No
	Visual	The bull was locked behind the gate	Yes
	McGurk fusion	The bull was locked behind the date	No

*The target word column indicates the conditions (e.g., sentence's meaning compatible with McGurk fusion and/or the unisensory inputs). The stimuli column indicates, respectively, what the input was in the auditory and visual channel and the expected perceived sentence; the column sentence provides an example. The column headed “Compatibility” indicates whether the final word was compatible or incompatible with the sentence context*.

Prior to the main experiment, these sentences and target word combinations were tested by an independent group of 12 young participants who were instructed to rate, using a 7-point Likert scale, the meaningfulness of each sentence. We also included filler sentences in this rating task for variety. The ratings from these independent judges confirmed our manipulations between sentence meaning and meaning of the target word. In order to assess whether in the completion of each of the sentences there was a bias to produce a given word, we also conducted a sentence completion test with another independent group of 8 participants. They were instructed to complete each of the sentences which was missing the final word. We then calculated how frequently the target word was produced as the final word in each sentence across all participants. The results for meaningful ratings and frequency of word associations are provided in Table 3 and further discussed in relation to the main study.

**Table 3:** **Mean rating of “meaningfulness” for the sentences in Experiment 2 in each condition**.

**Compatibility**	**Input**	**Mean “meaningfulness” ratings**		**Frequency of word association (number of target words/number of) produced words)**	
		***M***	***SD***	***M***	***SD***
McGurk only	Auditory	3.2	2.0	0	0
	McGurk	6.6	0.8	2	2.3
	Visual	1.9	1.0	0	0
McGurk and Auditory	Auditory	5.3	1.9	0	0
	McGurk	6.1	1.0	0.5	1.13
	Visual	2.8	2.0	0.2	0.6
McGurk and Visual	Auditory	2.2	0.7	1	2.3
	McGurk	5.1	1.7	0.5	1.1
	Visual	5.5	1.6	0	0
None	Auditory	1.7	0.8	0	0
	McGurk	1.6	0.4	0	0
	Visual	1.6	0.7	0	0
Visual-only	Auditory	2.4	1.3	3	2.8
	McGurk	2.1	0.4	0	0
	Visual	7.0	0.0	0	0
Auditory-only	Auditory	6.6	0.8	1.7	1.7
	McGurk	2.3	1.2	0	0
	Visual	1.9	1.3	0	0

*Mean frequency that the final word was spontaneously produced by independent judges per condition*.

#### Design

The design of the experiment was based on a Group (older vs. younger) by McGurk-fused response compatibility (sentence compatible or incompatible with the “McGurk” word) by compatibility with unisensory response (sentence compatible with the visual, or the auditory input or none of the unisensory components) mixed design. The Group factor was between-subjects whereas McGurk and unisensory compatibility factors were within-subjects factors. The dependent variable was the same as that described in Experiment 1, in that responses to the target word were classified as either “McGurk-fused,” McGurk-auditory, Mc-Gurk-viseme or “other.”

In the main experiment, one sentence was used as practice and all experimental conditions were presented based on manipulations of the target word in this sentence, yielding 6 practice sentences. The other 9 individual sentences were used as test stimuli (with six different versions of each based on each condition), yielding 54 test sentences in total. The presentation order of the sentences was randomized across participants.

#### Procedure

Participants were informed that they would be presented with sentences and their task was to repeat the sentence as they had understood it. The experimenter then recorded the sentence reported by participants.

### Results

**Figure 1:**
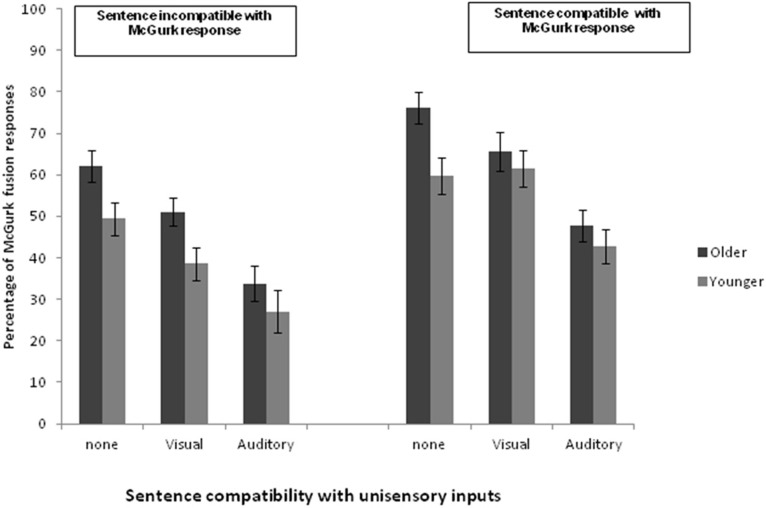
**Percentage of “McGurk-fused” responses per condition in Experiment 2.** The plots on the left (Sentence incompatible with McGurk response) represent participants' responses in both age groups when sentence meaning was not compatible with the fused word; on the right (compatible with McGurk response) the plots represent the same conditions when the sentence is compatible with the McGurk fusion word.

The final word of each reported sentence was categorized based on the same criteria as described in Experiment 1. The proportion of responses within each response category, which were dependent on the compatibility of the AV “McGurk” target word with sentence meaning or on each of the unisensory inputs (i.e., compatible with auditory or visual component), was calculated. These results are plotted in Figure 1. We then ran a 2 (compatible with the McGurk percept or not) × 3 unisensory compatibility (no unisensory compatibility or compatible with visual or compatible with auditory component) × 2 Group (older or younger group) mixed design ANOVA. A significant effect of group [*F*_(1, 24)_ = 5.99, *p* < 0.05] was found with older participants producing more “McGurk-fused” responses than younger participants (mean proportion of “McGurk-fused” responses were 0.56 and 0.46, respectively). There was also a main effect of compatibility with the McGurk fused word [*F*_(1, 24)_ = 58.79, *p* < 0.001] with, on average, more McGurk-fused responses produced when the meaning of the sentence was compatible with the McGurk response (0.59) than when it was not (0.44). Finally there was a main effect of unisensory compatibility [*F*_(1, 24)_ = 46.5, *p* < 0.001]: on average, more McGurk responses were produced when none of the unisensory inputs were compatible with sentence meaning (0.62) than when sentence meaning was compatible with either a visual (0.54) or auditory component (0.38; Newman–Keuls *post-hoc*, *p*s < 0.01). None of the interactions between the variables were significant.

When sentence meaning was compatible with both the McGurk fused word (i.e., the target word) and one of the unisensory inputs (either visual or auditory), the proportion of McGurk-fused response was always higher than the amount of auditory or visual based word responses [McGurk compared to Auditory responses in the “McGurk and Auditory” condition: *t* = 2.26, *p* < 0.05; McGurk compared to Visual responses in the “McGurk and Visual” condition: *t* = 11.77, *p* < 0.001]. This result confirms that, while participants were influenced by sentence meaning in responding, they were not entirely driven by it. If it were the case that sentence meaning drove the perception of the target word, then when the sentence was compatible with both the McGurk word and with one of its unisensory components, responses should have been roughly equally distributed between the McGurk-fused and the compatible unisensory response. Instead, we found that participants were responding consistently more accordingly to the (compatible) fused response than to the (compatible) unisensory input.

The results of the word association test lead to some limitations on this conclusion as it appears that the McGurk target word is more likely to be spontaneously associated with the sentence than either of the unisensory words. However, it is worth noting that despite this association, participants still responded by producing the unisensory word, even if it was weakly or not at all associated with the sentence, showing the relevance of the (manipulated) semantic context of the sentence, not of a spontaneous word association. In other words, when sentence meaning was compatible with either the visual or auditory inputs only, participants appeared to respond more in agreement with the meaning than with the unprompted word frequently associated with that sentence.

Considering that some intra- and inter-individual variability is to be expected with McGurk illusion word stimuli, we checked for the one to one correspondence between susceptibility to the illusion in Experiments 1 and 2 (condition where the A and V inputs are compatible with the McGurk fused response only) for the AV pair that we used in both experiments. All older adults showed a 100% by item correspondence, i.e., all items that produced a fused response in Experiment 1 also produced a fusion in Experiment 2 (with the addition of further items producing a fusion in Experiment 2 due to the semantic manipulation as expected). Ten out of thirteen younger adults also showed 100% correspondence and all showed correspondence equal or higher than 60%. A by item analysis between experiments on the average number of illusions in each group revealed a high correlation between experiments both in younger and older participants older *R*^2^ = 0.6, *p* = 0.02; younger *R*^2^ = 0.9, *p* < 0.01. Although these correlations have to be interpreted with caution due to the limited number of items available for comparison, they suggest a good reliability of the task across experiments.

### Discussion

In sum, while older participants were more susceptible than younger adults to the McGurk illusory responses, the effect of sentence meaning on the nature of the target word response (i.e., McGurk-fused, or response based on the auditory or viseme component) did not differ across the two groups.

Our results provided evidence that word perception in both groups was susceptible to the higher-level influence of semantic content of the sentences. However, older adults were more susceptible to the McGurk illusion than younger adults. We did not find a greater influence of context manipulation for older than for younger participants, suggesting that the difference between the age groups on susceptibility to the McGurk illusion was not due to the top-down influence of sentence meaning.

## General discussion

In the present study we found that older persons are more susceptible to the McGurk audio-visual speech illusion when words and words in sentences are presented than their younger counterparts and that susceptibility to the illusion is influenced but not entirely determined by semantic expectations in relation to meaning.

An age specific benefit of multisensory inputs in older compared with younger adults has been found in the literature when the task requires participants to rely more on one source of information than the other, either because the other has to be ignored, i.e., in selective attention tasks, (e.g., Poliakoff et al., 2006), or because the reliability of one source is higher than the other in some ways (i.e., in incongruent contexts), or else simply because one source provides information which is irrelevant to the task (e.g., background noise Hugenschmidt et al., 2009, see also Mozolic et al., 2010). In line with these considerations the present result shows that when auditory and visual inputs are incongruent, as it is the case in the McGurk illusion, older adults integrate these inputs more often than younger adults. An alternative explanation is that older adults pay more attention to the visual input in order to support their hearing (Thompson and Malloy, 2004), however, this explanation is not fully supported by the fact that no group difference was found in the visual only condition.

In Experiment 2, we found no interaction between the frequency of McGurk illusions experienced across younger and older groups and the susceptibility to context manipulations. The benefit of semantic compatibility (e.g., more auditory responses provided when the semantic content was congruent with the auditory input) did not differ significantly between younger and older participants. In both groups unisensory semantic biases were associated with a reduction in the number of illusions but not with their complete disappearance. In other words, even when the meaning of the sentence was compatible with either one of the unisensory inputs in the AV incongruent target word, participants were still susceptible to the McGurk illusion.

A limitation of this study is that our final sample of older adults is relatively highly educated, as lower educated elderly were excluded for the purpose of fair comparison with younger adults. Another limitation inherent to the use of audio-visual illusions is the relative inter-individual variability in the susceptibility to the illusion. In addition the relatively high frequency of responses falling in the Other category, due both to the conservative criterion we adopted in classifying the responses provided and the regional accent of the speaker also suggest that these results need to be replicated with a different set of stimuli to ensure their robustness. A further development could also aim to replicate the results with purely unisensory control conditions (e.g., the visual only signal is not accompanied by white noise). Nonetheless this study provides evidence that incongruent audio-visual words are merged more often by older than younger adults (Experiment 1) and this result occurs independently from top-down semantic biases that may favor the fused percept over its unisensory components (Experiment 2). The relationship between this kind of multisensory interaction and congruent language processing needs to the addressed in future studies.

Electrophysiological studies have shown that the McGurk illusion occurs at an early stage of signal processing (Saint-Amour et al., 2007). The left Superior Temporal Sulcus has been shown to play a crucial role in multisensory integration and in susceptibility to the McGurk illusion (Nath and Beauchamp, 2012). However, further studies are necessary to determine the level at which perceptual-semantic interactions occur.

At present, models that allow some contextual constraints on speech perception can account for these results because non-speech information such as visual information and higher level semantic constraints can contribute in recognizing an auditory input (Oden and Massaro, 1978; Massaro and Chen, 2008).

In conclusion the results of the present study suggest that, for the purpose of speech comprehension, older adults combine auditory and visual words more than younger adults, particularly when these words are composed by an incongruent combination of visual and auditory inputs. Importantly, we found in Experiment 2 that while both younger and older participants responded in accordance with semantic compatibility, older adults produced more McGurk illusion responses than younger adults irrespective of the nature of the relationship between sentence meaning and the compatible sensory component of the target word. This result supports the claim that perceptual more than higher level cognitive factors are at the grounds of the higher susceptibility to the McGurk illusion in older relative to younger adults found in the present study.

### Conflict of interest statement

The authors declare that the research was conducted in the absence of any commercial or financial relationships that could be construed as a potential conflict of interest.

## Appendix

**Table A1:** **List of items used in the study**.

**Audio**	**Visual**	**McG**
bait	gate	date
bale	cane	gale
bale	kale	gale
been	beep	beam
been	deep	beam
bent	dent	dent
bog	dog	dog
cap	calm	cat
cap	can	cat
clap	can	cat
cop	con	cot
grin	grip	grim
hip	hit	hit
lisp	list	list
mail	day	nail
map	mat	mat
nay	pay	may
neat	peat	meet
pale	tail	kale
pale	trail	kale
pea	tea	key
peek	tea	key
peep	tea	key
pill	tim	kin
pin	tin	kin
pram	cram	cram
ran	rap	ram
rip	rid	rig
shop	shot	shock
shop	shone	shot
veer	dear	gear
vet	get	debt
warn	warp	warm

*The acceptable fused responses were either the epected McGurk fusion or a word presenting an intermediate place of articulation between the visual and the auditory word or the same place of articulation as the visual word. Words other than the auditory word that presented the same place of articulation of the auditory word were classified as other*.
